# Response to: “Importance of method assumptions: Response to: “Challenges in undertaking nonlinear Mendelian randomization””

**DOI:** 10.1002/oby.24056

**Published:** 2024-05-21

**Authors:** KH Wade, NJ Timpson, FW Hamilton, N Sattar, D Carslake, G Davey Smith

**Affiliations:** 1 Medical Research Council (MRC) Integrative Epidemiology Unit, University of Bristol, Bristol, UK; 2 Population Health Sciences, Bristol Medical School, Faculty of Health Sciences, University of Bristol, Bristol, UK; 3 Infection Science, North Bristol NHS Trust; 4 Institute of Cardiovascular and Medical Sciences, British Heart Foundation Glasgow Cardiovascular Research Centre, University of Glasgow, Glasgow, UK

Dear Editors,

We read with interest the recent letter concerning our self-criticism^1^ of the non-linear Mendelian randomization (MR) methodology we applied in an earlier publication in this journal^2^. In a constructive critique, the authors challenge the suitability of assigned sex as a negative control outcome under the definition that a valid negative control outcome should not be plausibly related to the exposure (here, body mass index, BMI) and that these conditions can be tested within an analysis framework free of confounding and selection bias.

This is a reasonable point of consideration and one which justifiably cross-examines our use of the term “negative control”. However, this flags a more general issue – that our use of this term may not have been helpful from a formal point of view. Sadly, we fear that – again – the likely complications of non-linear MR as described in our recent perspective have been missed – that there are fundamental difficulties in the application of non-linear MR analyses and that current methods (including the doubly-ranked approach) are unable to avoid the generation of spurious associations.

We concede that the use of UK Biobank as a population sample is potentially imperfect (mostly due to the biased, volunteer-based sampling frame^3^) for the deployment of a pure negative control analysis, as it will for many other forms of both observational and applied epidemiological investigations. We have not provided an exhaustive simulation of the likely sources of possible bias to this extent nor have we undertaken the same analysis in multiple, independent studies (of different design) in efforts to quantify such flaws. However, what is important is that the analyses of both primary outcome and “negative control” (here, assigned sex) were undertaken in the same study and will suffer the many of the same problems whilst remaining implausibly related – BMI may well be related to assigned sex, but BMI does not cause the latter. Importantly, the magnitude of selection bias within this sample and specific question is unlikely to be large enough to explain these results and even if BMI was introducing the selection bias, this would influence the overall MR results as much as seen using non-linear methods, and most likely not in a different and incomprehensible manner^4, 5^.

There are a series of other potential arguments that would have been more well-placed targeting our own use of the phrase “negative control”. For example, we may well expect a variable claimed to be an “instrument” for BMI that is derived from a polygenic risk score^5^ to behave differently across strata of BMI (however formed), owing to the dimorphic characteristics of the underpinning genome-wide association study results. This is a potential biological flaw to the analysis and indeed one where the differences in stratum-specific estimates would be induced by the nature of relationship between “instrument” and negative control. However, even this – with anticipated properties – would not generate the clearly flawed results generated in the illustrative analyses. Critically, the undertaking of non-linear MR (even with new methods available which continue to generate questionable results^1, 6^), remains unreliable and are likely influenced by a host of features manifest with differing impact across studies (e.g., genetic architecture of exposures, outcome of interest, discovery genetic studies, distribution of exposure and relationship between exposure and outcome).

We welcome the authors’ overall point as to the need for the use of triangulation to ameliorate, or at least contextualise, inferential difficulties encountered in any study design. However, are still unconvinced that the refinements to analyses or wording are such to correct the problems flagged in our exemplar analysis from 2018 or to rescue existing methods attempting to undertake non-linear MR.
